# Occurrence of *Campylobacter* in Faeces, Livers and Carcasses of Wild Boars Hunted in Tuscany (Italy) and Evaluation of MALDI-TOF MS for the Identification of *Campylobacter* Species

**DOI:** 10.3390/foods12040778

**Published:** 2023-02-10

**Authors:** Monika Ziomek, Michał Gondek, Beatrice Torracca, Francesca Marotta, Giuliano Garofolo, Kinga Wieczorek, Katarzyna Michalak, Filippo Fratini, Francesca Pedonese

**Affiliations:** 1 Department of Food Hygiene of Animal Origin, University of Life Sciences in Lublin, Akademicka 12, 20-950 Lublin, Poland; 2 Department of Veterinary Sciences, University of Pisa, Viale delle Piagge 2, 56124 Pisa, Italy; 3 National Reference Laboratory for Campylobacter, Istituto Zooprofilattico Sperimentale dell’Abruzzo e del Molise “G. Caporale”, Via Campo Boario 1, 64100 Teramo, Italy; 4 National Veterinary Research Institute, Department of Hygiene of Food of Animal Origin, Partyzantow 57, 24-100 Pulawy, Poland; 5 Department and Clinic of Animal Internal Diseases, Faculty of Veterinary Medicine, University of Life Sciences, Głęboka 30, 20-612 Lublin, Poland; 6 Interdepartmental Research Center Nutrafood “Nutraceuticals and Food for Health”, University of Pisa, Via del Borghetto 80, 56124 Pisa, Italy

**Keywords:** *Campylobacter*, wild boar, food safety, MALDI-TOF MS, *C. lanienae*

## Abstract

A total of 193 wild boars hunted in Tuscany, an Italian region with a high presence of wild ungulates, were examined to assess the occurrence of *Campylobacter* species in faeces, bile, liver and carcasses, with the aim of clarifying their contribution to human infection through the food chain. *Campylobacter* spp. were found in 44.56% of the animals, 42.62% of the faecal samples, 18.18% of the carcass samples, 4.81% of the liver tissues and 1.97% of the bile samples. The *Campylobacter* species genotypically identified were *C. coli*, *C. lanienae*, *C. jejuni* and *C. hyointestinalis*. The prevalent species transpired to be *C. coli* and *C. lanienae*, which were isolated from all the matrices; *C. jejuni* was present in faeces and liver, while *C. hyointestinalis* only in faeces. Identification was carried out by matrix-assisted laser desorption/ionisation–time-of-flight mass spectrometry (MALDI-TOF MS) on 66 out of 100 isolates identified genotypically, and the technique yielded unsatisfactory results in the case of *C. lanienae*, which is responsible for sporadic human disease cases. The level of *Campylobacter* spp. contamination of meat and liver underlines the need to provide appropriate food safety information to hunters and consumers.

## 1. Introduction

Campylobacteriosis is a gastro-intestinal infection and the most reported zoonosis in the EU, with *Campylobacter jejuni* and *C. coli* as the main causative agents, and it represents a threat to public health worldwide [1,2,3]. It is usually self-limiting; however, in rare cases, and especially in immunocompromised individuals, it can lead to severe post-infection complications: arthropathies, immune-mediated neuropathy such as Guillain-Barré Syndrome, irritable bowel syndrome and septicaemia [4]. Although campylobacteriosis is mostly associated with the consumption of poultry meat and raw milk, *Campylobacter* spp. is also commonly found in the meat of other animal species [5].

The wild boar is currently the second most abundant ungulate in Europe, and it is considered a pest in many areas mainly because it damages crops, has a negative ecological impact and causes road traffic accidents [6,7]. Tuscany is the Italian region with the highest number of ungulates; it also has a higher ungulate density than all other European Union countries, except Austria [8]. In 2018 in Tuscany, the estimated ungulate population was 400,000 animals, with 40% represented by roe deer and 30% by wild boar. The estimated number of wild boars corresponded to 122,000 animals [8]. Wild boar hunting and meat consumption is traditionally widespread in many parts of Europe, including Italy. Moreover, in recent years, the most widely used and most effective method of mitigating the economic, ecological and agricultural impact of wild boars consists of programmes of culling by means of hunting [7,9]. These actions lead to an increase in wild boar meat availability on the market and its wider consumption.

Wild boars are also important reservoirs of infectious diseases, including campylobacteriosis [10]. Globally, the prevalence of *Campylobacter* in wild boars has been studied, with results demonstrating great variety in relation to the different geographical areas [11,12,13,14]. When wild boars are hunted, their meat is usually destined for human consumption, and since they are a reservoir for several *Campylobacter* species, including antimicrobial-resistant strains, they can represent a source of infection for humans [15].

Wild boar meat and offal, such as liver, are usually consumed after thorough cooking, of which *Campylobacter* spp. are unable to survive. There is, however, a risk associated with sausages containing wild boar meat which have not been properly ripened and, most importantly, with the potential cross-contamination of ready-to-eat foods during the preparation of cooked meals containing wild boar meat. There is also the possibility of a transmission of campylobacteriosis from wild boars to extensively reared livestock and vice versa [14]. This is particularly true in Tuscany, where highly appreciated autochthonous pig breeds such as the “Cinta Senese” are reared in a semi-wild breeding system that allows them to contact other wild animals unhindered. If infected, these animals could then introduce *Campylobacter* to the following stages of the food chain [16].

To obtain a reliable identification of the *Campylobacter* species involved in food-borne infections and alongside genotypic identification techniques (conventional and multiplex polymerase chain reaction (PCR), real-time quantitative PCR, 16S rDNA gene sequencing, multi-locus sequence typing, amplified fragment length polymorphisms [17,18]) and the most popularly used phenotypic identification methods (biotyping, serotyping, multilocus enzyme electrophoresis [19]), other rapid and accurate methodologies can be valuable tools. Matrix-assisted laser desorption/ionisation–time-of-flight mass spectrometry (MALDI-TOF MS) is nowadays a commonly used method to identify micro-organisms. MALDI-TOF MS utilization in food microbiology is constantly increasing due to several advantages. Indeed, testing requires small amounts of biomass and can be performed from primary culture. In addition, sample preparation is relatively simple, and MALDI-TOF MS analyses are timely and cost-effective [20,21]. Most data on its reliability, however, come from studies involving clinical or human strains, while the reliability for food-related *Campylobacter* has been less comprehensively studied, and then mainly with the focus on *C. jejuni* and *C. coli* [22,23].

The aim of this study was to evaluate the presence of *Campylobacter* in wild boars hunted in Italy in order to assess their role as a wild reservoir of this pathogen and the possibility of *Campylobacter* entering the food chain through wild boar meat and liver. Limited information is currently available concerning the presence of *Campylobacter* spp. in wild boar tissues intended for human consumption. To our best knowledge, this is the first report to isolate *C. lanienae* from wild boar liver and bile. We also tested the usefulness of MALDI-TOF MS in identifying *Campylobacter* isolates from wild boar (rare game-origin matrices) compared to the molecular standard. This was intended to alleviate the scarcity of MALDI-TOF *Campylobacter* data, especially regarding *C. lanienae*—one of the species most frequently isolated from wild boar of which the pathogenic potential for humans is still not fully known [24].

## 2. Materials and Methods

### 2.1. Animals

A total of 193 wild boars (111 females and 82 males; 87 adult, 30 sub-adult and 76 young animals) were sampled as part of a project focused on the isolation of pathogenic micro-organisms in hunted Tuscan wild boar, to study their role as a reservoir for human and animal diseases. The animals’ ages were determined based on tooth eruption and lower-jaw tooth wear [25]. Sampling took place during the autumn–winter hunting season from 2018 to 2019, following the regional hunting legislation [26] in 4 Tuscan provinces (Pisa, Livorno, Siena and Grosseto). Sampling was integrated with the routine procedure of delivery of shot animals (by the evening of the hunting day at the latest) to the central collection point, where evisceration and skinning operations were performed.

### 2.2. Sample Collection

The samples were taken by expert microbiology laboratory personnel during the standard course of action when hunted prey was dressed. Faecal swabs were obtained directly from the rectum using an Amies charcoal swab (Copan Italia S.p.A., Brescia, Italy). During the evisceration phase, bile was withdrawn from the gall bladder with a sterile syringe, and a sample of liver (about 50 g) was also collected. After the skinning, a Whirl-Pak Speci-Sponge (Nasco, Madison, WI, USA) (pre-moistened on site with 10 mL of sterile buffered peptone water) was used to sample the carcass tissue; a total area of approximately 300 cm^2^ for each half carcass from the belly/flank, medial thigh, and shoulder regions was sampled. Since the sampling was performed in collaboration with the hunters during regular hunting sessions, it was not always possible to collect all four types of samples from each animal examined. The total numbers of analysed samples were as follows: 183 faecal swabs, 187 liver tissue samples, 152 bile samples and 55 carcass sponges. All samples were transported to the laboratory under refrigerated conditions and analysed before 24 h had elapsed from the sampling time.

### 2.3. Sample Preparation and Campylobacter Enrichment

For all types of samples, enrichment was carried out in Bolton selective enrichment broth with the addition of the corresponding supplement (cefoperazone, vancomycin or trimethoprim, 20 mg/L in each case, and cycloheximide, 50 mg/L) and laked horse blood (5% *v*/*v*). For faecal samples, each swab was put in a tube with 9 mL of Bolton enrichment broth. For liver tissue, a swab was taken from a freshly cut surface exposed using a sterile scalpel and put in a tube with 9 mL of Bolton enrichment broth. Before the incision, the liver surface was sterilised by touching it with the red-hot blade of a scalpel to avoid external contamination [27]. For bile samples, 1 mL of sample was added to 9 mL of enrichment broth. Finally, for carcass samples, 70 mL of enrichment broth was added to each sponge bag, and the contents were mixed by squeezing the sponge repeatedly. All enrichment broths were incubated for 48 h at 42 ± 0.5 °C under microaerobic conditions using CampyGen sachets (5% O_2_, 10% CO_2_, 85% N_2_). The same incubation conditions were applied for all subsequent phases. All culture media, supplements and microaerobic sachets in all phases were acquired from Oxoid (Basingstoke, UK).

### 2.4. Campylobacter Isolation and Pre-Identification

*Campylobacter* isolation and preliminary identification at the genus level was carried out following the ISO 10272-1:2017 method modified by adding a supplemental filtration phase of the enrichment broth after the incubation, as described by Pedonese et al. [28,29]. Briefly, 0.3 mL of the enrichment broth was seeded onto a sterile cellulose membrane filter (47 mm diameter, 0.45 µm pore size, Sigma-Aldrich, Milan, Italy) laid on the surface of a modified charcoal cefoperazone deoxycholate agar (mCCDA) plate, and the filtrate was spread onto the agar surface. After incubation, 1 or 2 characteristic colonies per sample were picked from positive plates. Isolates were preliminarily identified at the genus level by using several phenotypic tests: microscope observation of cell morphology and motility with a contrast stain (1:1 Gram’s crystal violet, Pro-Lab Diagnostics, Bromborough, Wirral, UK, and saline solution) [30], an oxidase test (oxidase strips, Oxoid), a catalase test (hydrogen peroxide 3% solution, Merck Life Sciences S.r.l., Milan, Italy), growth trials (at 42 °C in aerobiosis and at 25 °C in microaerobic conditions) and with the Oxoid Biochemical Identification System Campy (Oxoid), a rapid identification system based on the detection of L-alanyl aminopeptidase production. Isolates phenotypically confirmed as *Campylobacter* spp. and fully viable were stored at −80 °C in brain–heart infusion broth with 5% laked horse blood and 15% glycerol (Sigma-Aldrich) for further analyses.

### 2.5. Molecular Identification

The isolates from positive samples were confirmed using a multiplex PCR as described by Wang et al. [31] and a simplex PCR according to Di Giannatale et al. [32], as previously reported [33]. For these analyses, DNA was extracted using the Maxwell 16-tissue DNA purification kit (Promega Corporation, Madison, WI, USA) according to the manufacturer’s instructions. In addition, isolates negative in PCR identification were subjected to 16S rRNA gene sequencing. Typing was performed by amplifying the 16S rRNA gene using the primers shown in Table 1 (Eurofins Genomics, Ebersberg, Germany), followed by sequencing of the PCR product. Amplification products were verified by gel electrophoresis. The products of the PCR were purified using ExoSAP-IT reagent (Applied Biosystems, GE Healthcare, Piscataway, NJ, USA) and sequenced using the BigDye Terminator v.3.1 Cycle sequencing kit (Applied Biosystems) according to the recommendations of the manufacturer. After sequencing, DNA was purified with ethanol precipitation using the Agencourt CleanSEQ^®^ kit (Beckman Coulter, Brea, CA, USA). Sequencing products were analysed with a Genetic Analyzer 3500^®^ (Life Technologies, Renfrewshire, UK). Basic local alignment search tool analysis of the partial 16S rRNA gene sequence obtained was performed using the taxonomy browser of the National Center for Biotechnology Information (www.ncbi.nlm.nih.gov, accessed on 6 December 2019).

### 2.6. MALDI-TOF MS Identification

**Figure 1:**
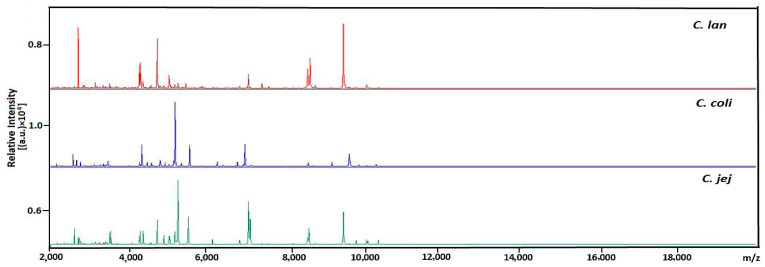
Characteristic spectra of *C. jejuni*, *C. coli* and *C. lanienae* isolates generated by the Bruker Ultraflextreme MALDI TOF system. The intensities and m/z of the ions are shown on the Y- and X-axis, respectively.

Sixty-six isolates were subjected to MALDI-TOF MS identification to verify the effectiveness of this technique in identifying *Campylobacter* species. The identification of bacterial isolates was preceded by preliminary extraction of proteins with ethanol and formic acid. For this purpose, a single colony of each bacterial culture was suspended in 150 μL of sterile deionized water, after which 450 μL of pure ethanol (Merck, Darmstadt, Germany) was added. Then, each sample was mixed thoroughly by vortexing. The resulting sample was then centrifuged for 5 min at 13,000 rpm. After that, the supernatant was discarded, 40 μL of 70% aqueous formic acid and then 40 μL of acetonitrile (Merck) were added to the precipitate, and the sample was thoroughly mixed by vortexing. After centrifugation (12,470× *g*), 1 μL of the obtained supernatant was pipetted onto a metal plate (Anchorchip 800 384, Bruker Daltonik, Bremen, Germany) and allowed to dry at room temperature. Then, 1 μL of cyano-4-hydroxycinnamic acid was added as the matrix solution (Bruker Daltonik), and the sample was left to dry at room temperature. The metal plate with the samples was subsequently placed in a MALDI chamber for analysis. An automatic measurement of the spectrum and a comparative analysis with reference spectra of bacteria were performed using the UltrafleXtreme mass spectrometer and MALDI-Biotyper 3.0 software (Bruker Daltonik). The analysis was performed in triplicate for each isolate. Mass spectra were recorded in active positive reflector mode within the 2000–20,000 mass-to-charge ratio (*m*/*z*) range using “complete sample” laser walk and 2000 shots per sample. The mass spectrometer automatically generated spectra of peaks corresponding to ions with different *m*/*z*, providing their number, intensity and peak correlation. Characteristic spectra for *Campylobacter* species generated by the Bruker UltrafleXtreme MALDI-TOF system were presented in Figure 1 and Figures S1–S3. Before identification, raw spectra were smoothed, and baseline was corrected and then compared with reference microbial spectra using MALDI-Biotyper 3.0 software with a database containing microbial profiles. The probability of correct identification in MALDI Biotyper 3.0 is expressed as a score index, and the following values indicate the level of identification: 2.300–3.000 is highly probable identification of the micro-organism to species level; 2.000–2.299 is highly probable identification of the micro-organism to genus level and likely identification to species level; 1.700–1.999 is likely identification to genus level; and 0–1.699 is unreliable identification. Using molecular diagnosis as the gold standard, the sensitivity and specificity of MALDI-TOF MS species identification was determined. The *Campylobacter* species identification was considered valid if the cut-off score reached or exceeded 2.0, referring to the database provided by Bruker Daltonics. The data were processed with Excel (Microsoft Corporation, Redmond, WA, USA).

## 3. Results and Discussion

Wild animals may represent a reservoir and source of various foodborne infections for humans, including campylobacteriosis. This disease can be transmitted indirectly by contaminated water, vegetables or domestic animals, or directly through contact with contaminated carcasses or by consuming raw or undercooked meat and meat products [34]. Previous studies have shown a significantly higher occurrence of *Campylobacter* spp. in wild boars as compared to other wild animals [15]. In this study, 44.56% (86/193) of the tested animals were found to be positive for *Campylobacter* spp. (Table 2).

A total of 100 isolates were genotypically identified at the species level. The presence of four *Campylobacter* species (*C. coli, C. lanienae*, *C. jejuni* and *C. hyointestinalis*) was registered by analysing wild boar faecal swabs, liver tissue, bile, and carcass samples (Table 3). *Campylobacter* spp. were found in 42.62% (78/183) of the faecal samples tested, of which 50% (39/78) were identified as *C. coli*, 41.03% (32/78) as *C. lanienae*, 5.13% (4/78) as *C. hyointestinalis* and 3.85% (3/78) as *C. jejuni*. Of the liver samples, 4.81% (9/187) were positive for *Campylobacter* spp.; *C. coli*, *C. jejuni* and *C. lanienae* were found evenly at 33.33% (3/9) of each species. In the tested bile samples, a prevalence of only 1.97% (3/152) was observed for *Campylobacter,* and the following species were confirmed: *C. coli* in 33.33% (1/3) and *C. lanienae* in 66.66% (2/3) of specimens. Regarding carcasses, 18.18% (10/55) of samples were positive for *Campylobacter*; *C. coli* and *C. lanienae* were found in 50% (5/10) of these contaminated carcasses.

Reviewing global studies, the *Campylobacter* rate of positivity and species diversity in wild boar varied significantly depending on the geographical areas where the animals came from and the type of sample tested. In Swedish wild boar, Wahlström et al. reported 10.6% *Campylobacter* positivity in faecal samples. In Switzerland, *Campylobacter* was not found in the faecal samples or the tonsils of 153 wild boar [11,12]. More recently, Tomino et al. reported 12.5% positive faecal samples among 248 wild boars hunted in Japan, with *C. hyointestinalis* as the most represented species; however, *C. lanienae* and *C. coli* were also confirmed in this study [35]. Generally, a high rate of *Campylobacter* prevalence in wild boar faecal samples was reported in Spain: 66% of 287 samples in southern central Spain, with prevalence ranging from 33% to 100% depending on the hunting region, and 38.9% of 126 samples in southern Spain, with most isolates being *C. lanienae* (*n* = 34) [13]. However, *C. coli* (*n* = 8) and *C. jejuni* (*n* = 2) were also confirmed [11]. In the same period, Navarro-Gonzalez et al. reported 12% of samples positive for *Campylobacter* bacteria presence out of 150 tested in north-eastern Spain, with a higher prevalence (24.6%) when sample enrichment was performed [12]. In this case, *C. lanienae* was also found as the most prevalent *Campylobacter* species. A very high prevalence (54.6%) of *Campylobacter* was reported in the faeces of wild boar in the Czech Republic, with *C. coli* as the predominant species (46.9%), followed by *C. jejuni* (13.4%) [36]. It should also be stated that studies regarding contamination of wild boar carcasses with *Campylobacter* are limited. A low carcass contamination level was reported in Germany, with only two positive samples for *C. coli* (1.57%) and one for *C. jejuni* (0.79%) out of 127 carcasses examined [37].

Similarly, data on the occurrence of *Campylobacter* spp. in Italian wild boar are scant. Ercolini et al. tested 50 muscle tissue samples from wild boars that were collected in Liguria and reported that only 2% were *Campylobacter*-positive (for *C. lari*) [38]. More recently, Stella et al. studied the presence of *Campylobacter* in wild boar hunted in northern Italy, and found 51.8% of faecal samples positive out of 56 tested and 16.7% of carcasses positive out of 30, with absolute values similar to those obtained in our study, in which 42.62% of faecal samples and 18.18% of carcasses samples were positive [39].

Our studies also showed the presence of *Campylobacter* in the liver and gallbladder of examined wild boar. Hepatic contamination with *Campylobacter* was proven in poultry, where it was a not-uncommon cause of human food-borne disease strongly linked to the ingestion of undercooked pâté [40]. Previous research also investigated these bacteria in suids: The prevalence of *Campylobacter* in pig livers was studied in Germany by von Altrock et al., and 10% of the 1500 pig livers sampled from 50 fattening herds were found to be positive, with *C. coli* as the predominant species [41]. Also in the UK, *Campylobacter* was isolated from 18.3% of pig offal samples, including liver, drawn from retailed food [42]. It should be stated, however, that new data regarding the occurrence of *Campylobacter* in wild boar liver are only gathered sporadically [43], and the first confirmation that raw liver may represent an important source of infection for the consumer or the operators who eviscerate wild boar after hunting is provided in our results. It should also be emphasised that even though *C. lanienae* has often been isolated from wild boar [44], this is the first time that this species has been isolated from wild boar liver and bile.

The present study confirmed multiple infections involving two species of Campylobacter in four tested wild boar. The possibility of a co-infection with different strains and species of *Campylobacter* was previously highlighted both in animals and humans [45,46,47]. The simultaneous occurrence of several *Campylobacter* species was confirmed in dog faeces samples where carriages of *C. coli/C. lari, C. jejuni/C. coli, C. jejuni/C. upsaliensis* and *C. upsaliensis/C. lari* were observed [48]. In humans, co-infection with multiple *Campylobacter* was described in infants co-infected with *C. jejuni/C. infans/C. curvus/C. concisus/C. helveticus/C. upsaliensis, C. jejuni/C. infans/C. upsaliensis* and *C. concisus/C. infans* species [49].

Our study also indicates a higher prevalence of *Campylobacter* in males (25.39%) than in females (19.17%). This phenomenon is difficult to explain; however, in this context, Zeng et al. demonstrated a more efficient immune response to Gram-negative bacteria in female than in male mice [43]. It was proven that innate antibodies against Gram-negative bacteria, such as enteropathogenic *Escherichia coli*, were present only in female mice, developed as a response to oestrogen hormones, and were not dependent on previous exposure to the antigen [50]. Thus, differences in sex hormone levels could play a significant role in the immune response to bacterial infection and partially explain the results obtained in this study. This correlation was also supported by human *Campylobacter* incidence studies where, in meta-analysis, the data confirmed higher incidence rates of campylobacteriosis in men than in women [51]. Our study also proved that young animals can be an important source of *Campylobacter*, hosting larger bacteria populations than sub-adult and adult individuals. In fact, there is little information in the global literature about age-based changes in gut microbiota composition in wild boar. It is known, however, that the process of *Campylobacter* gastrointestinal tract colonisation in chickens may be affected by the maturation stage of the immune system and the developmental stage of the gut microbiota composition [52,53]. In studies conducted on pigs, it has also been indicated that Proteobacteria (to which *Campylobacter* belongs) are relatively more abundant in the guts of piglets, but their relative abundance considerably decreases in adulthood [54].

Recently, MALDI-TOF MS was highly recommended for *Campylobacter* spp. isolates identification and was considered an accurate alternative to traditional identification methods [55,56]. In the present study, out of 66 examined isolates (Table 4), 93.94% (62/66) were identified to the genus level as *Campylobacter* spp., with scores of ≥1.700, and only 6.06% (4/66) scored <1.700 (all four belonged to the *C. lanienae* species). To the species level, in the case of *C. coli* isolates, 20.00% (7/35) were confirmed with scores of ≥2.300, 70.00% (26/35) had scores in the range of 2.000 to 2.299, and only 5.71% (2/35) had scores in the range of 1.700 to 1.999. In the case of *C. jejuni*, 100.00% (2/2) of the isolates were confirmed, with scores of 2.000–2.299. Contrastingly, *C. lanienae* isolates were identified considerably less conclusively, with 13.79% (4/29) yielding scores <1.700 and categorised as unreliable identification, 51.72% (15/29) scoring between 1.700 and 1.999 as identification only to the genus level, and only 34.48% (10/29) having scores of 2.000–2.299; also, no isolates were identified as this species with the highest scores in the 2.300–3.000 range. The *Campylobacter* species identification for *C. coli* by MALDI-TOF MS yielded sensitivity at 0.94 (0.814–0.984) and specificity at 1.00 (0.890–1.00) (Table 5). Regarding *C. jejuni*, sensitivity was 1.00 (0.342–1.00) and specificity was 1.00 (0.943–1.00). The sensitivity attained for *C. lanienae*, however, was no greater than 0.344 (0.199–0.526), whereas the specificity reached 1.00 (0.905–1.00).

The successful identification of micro-organisms using MALDI-TOF MS mainly relies on the database containing the appropriate spectra of known organisms and on the obtainment of a sufficient protein signal [20]. The level (score) of identification is determined by existing mass-spectral signature patterns in the database and factors which influence spectra quality, especially culture conditions such as the type of media used and the atmosphere in which bacterial isolates are grown, technical issues such as the age and intensity of the laser and the number of laser shots applied, as well as the type of MALDI-TOF MS target plates and matrix used [57]. It has been proven that for infrequently encountered microorganisms, the results of MALDI-TOF MS identification are reliable if the protein spectra of such micro-organisms are covered by the database; otherwise, the generated results may only give a hint by finding a related species or, in some cases, may misidentify the micro-organism as a very closely related species [58,59]. *Campylobacter lanienae*, for which the lowest scores were given, is not often identified to the species level using the MALDI-TOF technique, and this species is also often overlooked in food safety and animal health diagnostic protocols. Therefore, the database used may contain a lower number of reference spectra for *C. lanienae*, and the paucity of data could explain the lower score values for this species that were obtained in this study. There is a need to extend the databases with the spectra from different *C. lanienae* strains, and particularly those isolated from various game-origin food matrices. Recently, the reliability of MALDI-TOF MS for *Campylobacter* identification was tested on 27 strains of *C. coli* and *C. jejuni* isolated from birds and gave 100% correct results [56]. On a tenfold higher number of *Campylobacter* isolates from poultry and turkey products, MALDI-TOF MS identified *C. jejuni*, *C. coli* and *C. lari* 96% correctly overall [59].

As for other *Campylobacter* species commonly reported in wild boar, such as the *C. lanienae* evident in this study, few data are available on the usefulness of MALDI-TOF MS for their identification. Kérouanton et al. used MALDI-TOF MS to identify isolates of *C. lanienae,* although they did not report data on the reliability and quality of the identification or whether some strains were not identified [60]. Identifying the species of the strain involved in the first case in Europe of *C. lanienae*-related human gastroenteritis, Fornefett et al. reported a low MALDI-TOF MS identification score and only confirmed the isolate identification by 16S rRNA gene partial sequencing and species-specific PCR [61]. It was shown for the first time using MALDI-TOF MS that it is possible to obtain secure species identification for some *C. lanienae* isolates. This study shows reliable identification scores for 34% of the tested *C. lanienae*. Making a correct identification of *C. lanienae* should not be overlooked, as its actual pathogenic potential is still not fully known. In fact, besides the first European case of gastroenteritis with this aetiological agent in a butcher from Germany investigated by Fornefett et al. (which was mentioned previously), there is also on record the report of the first human case of enteritis caused by *C. lanienae* in a 39-year-old Canadian woman living on a pig farm [24,61]. The microorganism has also been isolated in slaughterhouse workers in Switzerland [62].

## 4. Conclusions

The present study shows that wild boar may be a conspicuous source of *Campylobacter* spp., because 44.56% of the tested animals were found to be positive. It should be emphasised that all identified species of *Campylobacter*, i.e., *C. coli*, *C. lanienae*, *C*. *jejuni* and *C. hyointestinalis*, are a public health concern. The *Campylobacter* spp. on carcasses and offal may be a direct source of human infection, especially for hunters, game dressers, butchers and game consumers, while *Campylobacter* in faecal samples may implicate faecal contamination of the environment in indirect zoonotic transmission. It was shown that MALDI-TOF MS combined with a reliable database could be a powerful method for the identification of *C. coli* and *C. jejuni* species (the species of most significant epidemiological importance and those frequently identified). Taking into account the achieved *C. lanienae* MALDI-TOF MS identification parameters, it should be stated that there is a need to constantly extend spectrum databases in order to increase the sensitivity of the method.

There are some limitations in our research. Firstly, it was not always possible to collect all four types of samples for each wild boar individual. In addition, not all collected isolates were confirmed using MALDI-TOF because of the loss of their vitality. Despite these problems, we conducted an in-depth analysis of *Campylobacter* infections in wild boar, taking into account the sampling site, the animal age and its sex. We contended that MALDI-TOF MS is a useful tool for major *Campylobacter* species identification and showed the limitations of this method in identifying a minor species such as *C. lanienae*. The investigation draws attention to this species, which is frequently found in wild boar and has neglected but not totally negligible pathogenic potential. The occurrence of *Campylobacter* in wild boar faeces, meat and offal requires communication measures targeted to hunters and consumers and intended to minimize the possibility of food-borne disease outbreaks linked to the improper handling of hunted animals and their meat.

## Figures and Tables

**Table 1:** Primers used for 16S rRNA gene sequencing.

Primers	Sequence (5′–3′)
16S_F517	5′-GCC AGC AGC CGC GGT-3′
16S_R517	5′-AAG GAG GTG ATC CAG-3′

**Table 2:** Prevalence of genotypically confirmed *Campylobacter* in all tested wild boars (*n* = 193) and in the wild boars harbouring genotypically confirmed *Campylobacter* (*n* = 86), categorized by sex and age.

	Total	% of 193 *	% of 86 **
Male	49	25.39	56.98
Female	37	19.17	43.02
Adult	34	17.62	39.54
Subadult	11	5.70	12.79
Young	41	21.24	47.67

193 *: total number of wild boars tested in this study; 86 **: total number of wild boars harbouring genotypically confirmed *Campylobacter.*

**Table 3:** Identity, sex, and age group of wild boars harbouring genotypically confirmed *Campylobacter*.

Animal Id.	Sex	Age Group	Faecal Swab	Liver	Bile	Carcass
Wb3	F	A	*C. lan ^#^*	−	−	\
Wb4	F	Y	*C. lan ^#^*	*	−	\
Wb18	F	SA	−	*C. lan ^#^*	−	\
Wb24	F	Y	*C. coli ^#^*	\	\	\
Wb27	M	A	*C. lan ^#^*	−	\	\
Wb28	M	A	*C. coli ^#^*	−	−	*C. coli*
Wb29	M	SA	*C. lan ^#^*	−	\	\
Wb32	M	A	*C. coli ^#^*	−	−	*C. coli*
Wb35	F	SA	*C. coli ^#^*	−	−	\
Wb36	F	A	*C. coli ^#^*	*C. coli*	−	\
Wb37	F	Y	*C. jej ^#^*	−	−	\
Wb38	F	A	*C. coli ^#^*	−	−	\
Wb39	F	Y	*C. coli ^#^*	−	−	\
Wb44	F	A	\	*C. coli ^#^*	\	\
Wb49	F	Y	−	−	−	*C. coli ^#^*
Wb55	M	Y	*C. coli*	−	−	\
Wb57	M	A	*C. lan ^#^*	−	−	*C. lan ^#^*
Wb59	F	A	*C. lan*	−	−	−
Wb67	F	A	*C. lan*	*C. jej ^#^*	−	\
Wb75	M	SA	*C. lan ^#^*	−	−	−
Wb76	F	Y	*C. coli ^#^*	−	−	−
Wb77	M	Y	*C. coli ^#^*	−	−	−
Wb78	M	Y	*C. coli ^#^*	−	\	*C. coli ^#^*
Wb85	F	A	*C. coli ^#^*	−	−	\
Wb98	F	Y	*C. coli ^#^*	−	−	*C. lan ^#^*
Wb101	F	A	*C. lan ^#^*	−	−	−
Wb103	M	Y	*C. lan*	−	−	*C. lan*
Wb107	F	A	*C. lan ^#^*	−	\	\
Wb108	F	A	*C. lan ^#^*	−	−	\
Wb109	F	A	*C. coli ^#^*	−	−	\
Wb110	F	SA	*C. lan ^#^*	−	−	\
Wb111	F	A	*C. coli ^#^*	−	−	\
Wb112	M	Y	*C. coli ^#^*	−	−	\
Wb113	F	A	*C. coli ^#^*	−	−	\
Wb115	F	Y	*C. lan ^#^*	−	−	\
Wb116	M	Y	*C. lan ^#^*	−	−	\
Wb118	M	Y	*C. lan*	−	−	\
Wb125	F	A	*C. lan ^#^*	−	*C. lan ^#^*	\
Wb126	F	A	*C. lan*	−	−	\
Wb128	M	Y	*C. lan ^#^*	−	−	\
Wb129	F	Y	*C. lan ^#^*	−	−	\
Wb130	M	A	*C. lan ^#^*	−	−	\
Wb133	M	A	*C. coli ^#^*	−	\	\
Wb134	M	A	*C. coli ^#^*	−	−	\
Wb136	F	A	*C. coli ^#^*	−	−	\
Wb137	F	Y	*C. coli ^#^*	−	−	\
Wb138	F	A	*C. coli ^#^*	−	−	\
Wb139	F	Y	*C. coli ^#^*	−	−	\
Wb140	F	A	*C. coli ^#^*	−	−	\
Wb141	F	Y	*C. coli ^#^*	−	−	\
Wb142	F	Y	*C. coli*	−	−	\
Wb143	M	Y	*C. coli*	−	−	\
Wb144	M	Y	*C. coli*	*C. coli*	\	\
Wb145	M	Y	*C. coli ^#^*	*C. lan ^#^*	−	\
Wb146	M	Y	*C. coli ^#^*	\	\	\
Wb147	M	A	−	−	−	*C. lan*
Wb148	M	SA	*C. lan ^#^*	−	\	\
Wb151	F	A	*C. lan ^#^*	−	−	−
Wb152	M	A	*C. jej*	−	\	\
Wb153	M	A	*C. jej*	−	\	\
Wb155	F	A	*	*C. jej*	−	\
Wb156	M	Y	*	*C. jej*	−	\
Wb157	F	Y	*C. coli*	−	−	\
Wb159	M	A	−	−	*C. lan*	\
Wb162	M	Y	*C. coli*	−	−	−
Wb164	M	Y	*C. coli*	−	−	−
Wb171	M	A	*C. coli*	−	−	−
Wb172	M	SA	*C. coli*	−	−	\
Wb173	F	Y	*C. lan*	−	−	\
Wb174	F	SA	*C. lan*	−	−	\
Wb175	F	Y	*C. lan*	*	−	\
Wb176	F	Y	*C. lan*	−	−	\
Wb180	F	SA	*C. lan*	−	\	\
Wb182	M	A	*C. hyo*	−	−	\
Wb183	F	A	*C. hyo*	−	\	\
Wb186	M	SA	*C. lan ^#^*	−	−	\
Wb189	M	Y	*C. lan ^#^*	−	−	−
Wb191	F	Y	*C. coli ^#^*	−	−	−
Wb192	M	Y	*C. hyo*	−	−	−
Wb193	F	Y	*C. lan ^#^*	−	\	*C. lan ^#^*
Wb194	F	Y	*C. coli ^#^*	−	−	*C. coli ^#^*
Wb195	F	Y	*C. coli ^#^*	−	−	\
Wb197	M	Y	*	*C. lan ^#^*	*C. coli ^#^*	−
Wb198	F	Y	*C. hyo*	−	−	\
Wb199	F	Y	*C. coli ^#^*	−	−	\
Wb200	M	SA	*C. lan ^#^*	−	−	−
Total	86	86	78	9	3	10

Animal id.: wild boar identification number; F: female; M: male; A: adult; SA: subadult; Y: young; *C. hyo*: *C. hyointestinalis*; *C. jej*: *C. jejuni*; *C. lan*: C. lanienae, *: presence of a positive isolate for *Campylobacter* spp. which was lost before genotyping; −: negative for *Campylobacter* spp.; \: sample not available., *^#^* isolates intended for MALDI-TOF MS identification.

**Table 4:** Identification of *Campylobacter* isolates (*n* = 66) by matrix-assisted laser desorption/ionisation–time-of-flight mass spectrometry (MALDI-TOF).

Species	No. of Isolates	No. of Isolates with Id Score < 1.700	No. of Isolates with Id Score 1.700–1.999	No. of Isolates with Id Score 2.000–2.299	No. of Isolates with Id Score 2.300–3.000
*C. coli*	35	0	2	26	7
*C. jejuni*	2	0	0	2	0
*C. lanienae*	29	4	15	10	0

**Table 5:** MALDI-TOF identification results of tested *Campylobacter* isolates (*n* = 66) (with genotypic identification taken to be authoritative).

	*C. coli* *n* = 35	*C. jejuni* *n* = 2	*C. lanienae* *n* = 29
Comparisons	35	2	29
NR	2	0	19
Correctly identified	33	2	10
Not correctly identified	0	0	0
Misidentified	0	0	0
Sensitivity (95% CI)	0.942 (0.814–0.984)	1.00 (0.342–1.00)	0.344(0.199–0.526)
Specificity (95% CI)	1 (0.890–1.00)	1 (0.943–1.00)	1 (0.905–1.00)

NR: results which were not reliable—MALDI-TOF score ≤2.00; Correctly identified: MALDI-TOF score ≥2.00; Not correctly identified: the indicated species was identified by MALDI-TOF as a different one; Misidentified: a different species was identified by MALDI-TOF as the indicated one; 95% CI: 95% confidence interval.

## Data Availability

The data supporting reported results are available from the corresponding authors upon request.

## Supplementary Materials

The following supporting information can be downloaded at: https://www.mdpi.com/article/10.3390/foods12040778/s1, Figure S1: Characteristic spectra of *Campylobacter coli* isolates (*n* = 35) generated by the Bruker Ultraflextreme MALDI TOF system. The intensities and m/z of the ions are shown on the Y- and X-axes, respectively. Sample codes (in accordance with those in Table 3) are displayed on the right at the end of each spectrum; Figure S2: Characteristic spectra of *Campylobacter lanienae* isolates (*n* = 29), generated by the Bruker Ultraflextreme MALDI TOF system. The intensities and m/z of the ions are shown on the Y- and X-axes, respectively. Sample codes (in accordance with those in Table 3) are displayed on the right at the end of each spectrum; Figure S3: Characteristic spectra of *Campylobacter jejuni* isolates (*n* = 2) generated by the Bruker Ultraflextreme MALDI TOF system. The intensities and m/z of the ions are shown on the Y- and X-axes, respectively. Sample codes (in accordance with those in Table 3) are displayed on the right at the end of each spectrum.
