# Supplementary material for: Occurrence of Campylobacter in Faeces, Livers and Carcasses of Wild Boars Hunted in Tuscany (Italy) and Evaluation of MALDI-TOF MS for the Identification of Campylobacter Species
Source: Foods. 2023 Feb 10;12(4):778. doi: 10.3390/foods12040778 (PMC9956588; doi:10.3390/foods12040778)
Supplement: Supplementary file 1 [file foods-12-00778-s001.zip › foods-2125825-supplementary.pdf]

Supplementary material

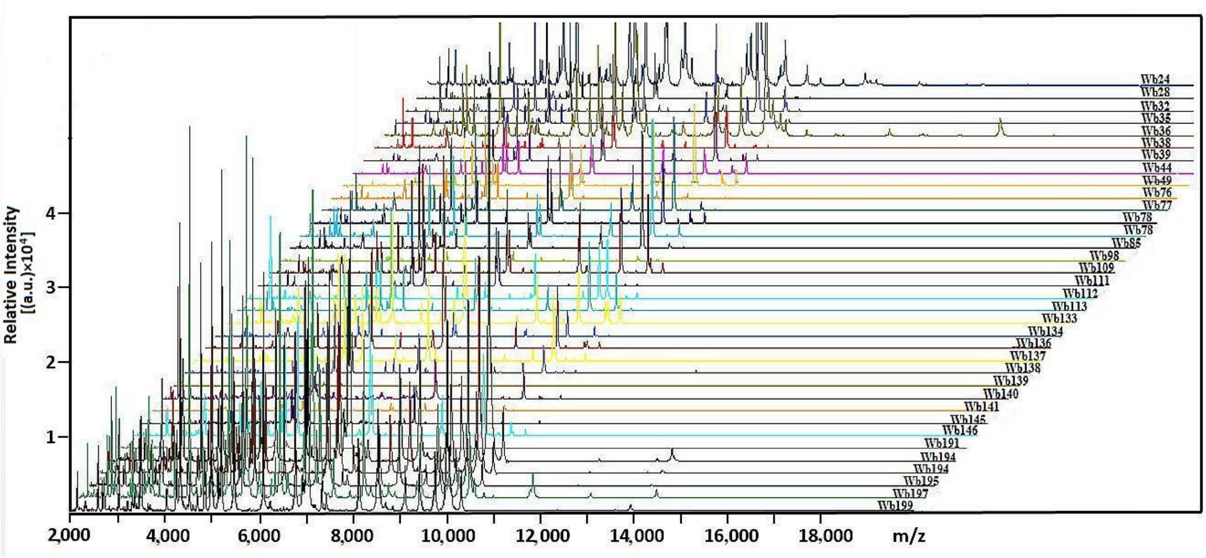

Figure S1. Characteristic spectra of *Campylobacter coli* isolates (n=35) generated by the Bruker Ultraflextreme MALDI TOF system. The intensities and m/z of the ions are shown on the Y- and X-axes, respectively. Sample codes (in accordance with those in Table 3) are displayed on the right at the end of each spectrum.

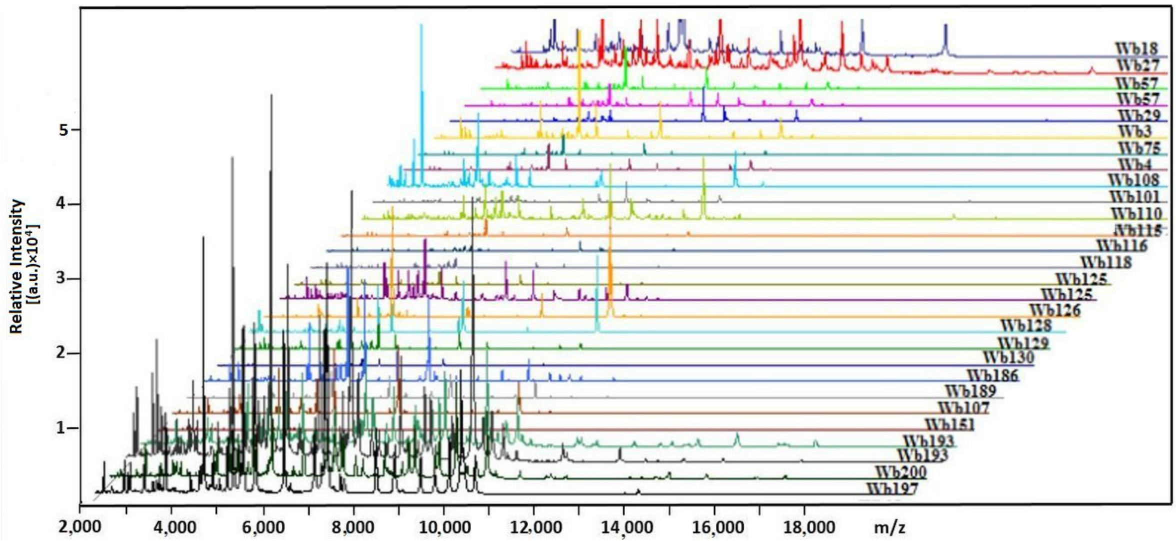

Figure S2. Characteristic spectra of *Campylobacter lanienae* isolates (n=29), generated by the Bruker Ultraflextreme MALDI TOF system. The intensities and m/z of the ions are shown on the Y- and X-axes, respectively. Sample codes (in accordance with those in Table 3) are displayed on the right at the end of each spectrum.

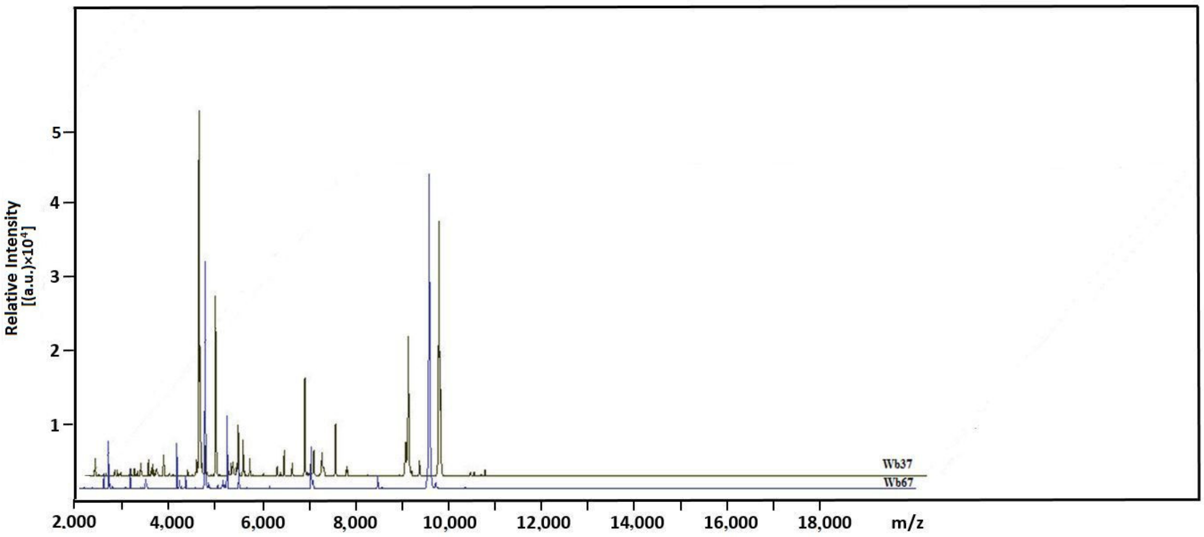

Figure S3. Characteristic spectra of *Campylobacter jejuni* isolates (n=2) generated by the Bruker Ultraflextreme MALDI TOF system. The intensities and m/z of the ions are shown on the Y- and X-axes, respectively. Sample codes (in accordance with those in Table 3) are displayed on the right at the end of each spectrum.
